# The Axial Alignment of Titin on the Muscle Thick Filament Supports Its Role as a Molecular Ruler

**DOI:** 10.1016/j.jmb.2020.06.025

**Published:** 2020-08-07

**Authors:** Pauline Bennett, Martin Rees, Mathias Gautel

**Affiliations:** The Randall Centre for Cell & Molecular Biophysics, School of Basic and Medical Biosciences, New Hunt's House, Guy's Campus, King's College London, London, UK

**Keywords:** MyBP-C, myosin binding protein-C, MyBP-H, myosin binding protein-H, CSR, C-zone super-repeat, DSR, D-zone super-repeat, AP, accessory protein, STORM, STochastic Optical Reconstruction Microscopy, STED, STimulated Emission Deletion microscopy, MyBP-C, MyBP-H, STORM, STED

## Abstract

The giant protein titin is expressed in vertebrate striated muscle where it spans half a sarcomere from the Z-disc to the M-band and is essential for muscle organisation, activity and health. The C-terminal portion of titin is closely associated with the thick, myosin-containing filament and exhibits a complex pattern of immunoglobulin and fibronectin domains. This pattern reflects features of the filament organisation suggesting that it acts as a molecular ruler and template, but the exact axial disposition of the molecule has not been determined. Here, we present data that allow us to precisely locate titin domains axially along the thick filament from its tip to the edge of the bare zone. We find that the domains are regularly distributed along the filament at 4-nm intervals and we can determine the domains that associate with features of the filament, such as the 11 stripes of accessory proteins. We confirm that the nine stripes ascribed to myosin binding protein-C are not related to the titin sequence previously assumed; rather, they relate to positions approximately 18 domains further towards the C terminus along titin. This disposition also allows a subgroup of titin domains comprising two or three fibronectin domains to associate with each of the 49 levels of myosin heads in each half filament. The results strongly support the role of titin as a blueprint for the thick filament and the arrangement of the myosin motor domains.

## Highlights

• Titin domains were localised in muscle A-bands by STORM and STED microscopy.
• Titin A-band domains uniformly span the cross-bridge region of the thick filament.
• The titin domains that colocalise with accessory protein stripes are determined.
• The titin domain pattern matches the 49 levels of myosin in the half filament.
• The role of titin as a blueprint for the thick filament is strongly supported.

## Graphical abstract

**Figure:**
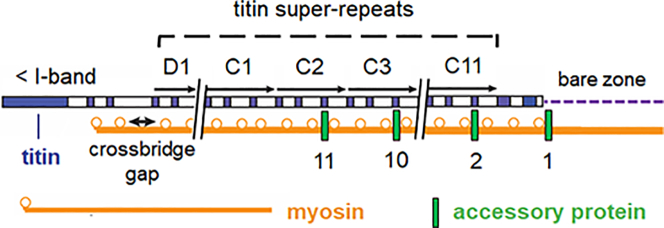

## Introduction

The thick filament of vertebrate striated muscle exhibits a precise and complex structure. On each side of a central bare zone, 49 levels of myosin heads or cross-bridges are arrayed at 14.5nm intervals [1]. Two main regions can be distinguished in these cross-bridge zones. The most prominent feature is an arrangement of 11 transverse stripes of accessory proteins (AP#1–#11) spaced at ~ 43nm intervals and seen in each half of the A-band starting at the edge of the bare zone [[2], [3], [4]]. Of these, the nine stripes distal from the bare zone are ascribed to the myosin binding protein family, in particular myosin binding protein-C (MyBP-C) [[5], [6], [7]]. This region of the filament has been termed the C-zone [8]. The outer 35% of the filament, the unstriped distal region (D-zone), is characterised by a gap in the cross-bridge repeat, two cross-bridges from the end of the filament [9]. The giant protein titin is thought to act as a molecular ruler and template for the thick filament [[10], [11], [12]], but its arrangement on the filament is not accurately known and is the subject of this study.

**Figure 1:**
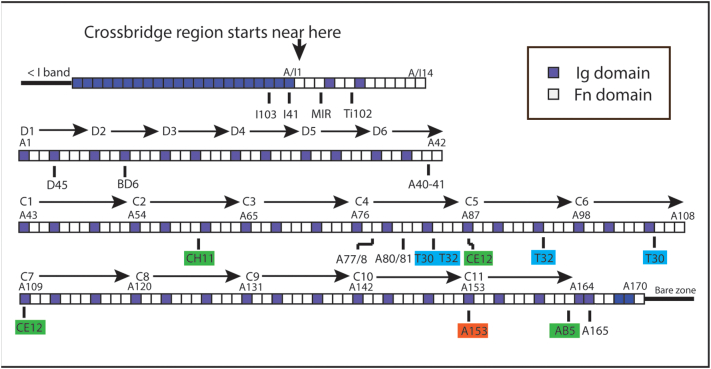
The pattern of Ig and Fn domains of thick filament-associated titin [16]. The convention of labelling the A-band domains sequentially with the nomenclature taken from Labeit and Kolmerer [16] and Granzier *et al*. [17] is used. An alternative is to label the Fn and Ig domains separately in order from the N- to C-terminal of titin. A comparison of the two can be found in Supplementary Table 1. More information on individual domains is to be found on the TITINdb website [18] (http://fraternalilab.kcl.ac.uk/TITINdb). The sequence associated with the cross-bridge region of the filament includes the 14 A/I junction domains and the 170 domains A1–A170, which cover the 6 D super-repeats of 7 domains (D1–D6) and 11 C-zone super-repeats of 11 domains (C1–C11) indicated. The first Ig domain of each C-zone super-repeat is numbered. Titin antibodies and their target domains are indicated below the sequence. See Table 1, Table 2 for more information. Those not highlighted are from published data. Red highlight is for a new antibody used in this study. Green highlighted are antibodies raised by John Trinick and further characterised here [10]. Blue highlighted are antibodies raised by Fürst *et al*. [21], whose epitopes have been determined here.

Titin is essential for the organisation, activity and health of vertebrate striated muscle [13]. It spans half a sarcomere from the Z-disc to the M-band [14], with the N-terminal portion running through the I-band and acting as an elastic element [15]. The C-terminal portion of the protein is closely associated with the thick, myosin-containing filament and consists of a series of ~ 200 immunoglobulin type 2 (Ig) and fibronectin type 3 (Fn) domains (Figure 1). A study to map the portion of titin associated with the ends of the thick filament found the first Fn domains of the sequence (A/I1–14 in Figure 1, nomenclature Granzier *et al*. [17]) to correspond to the first myosin cross-bridge levels at the tips of the filament [22]. Towards the centre of the filament beyond the final Fn domain (A170), an Ig-rich C-terminal sequence has been associated with the bare zone, where the ends of the myosin tails associate in an antiparallel arrangement [27].

Between these extremes, the Fn-rich region mainly comprises subgroups of one Ig domain followed by two or three Fn domains (Ig2Fn and Ig3Fn) (Figure 1). These 170 domains, A1–170 in the notation of Labeit and Kolmerer [16], comprise 6 distal (D) super-repeats each of 7 domains (DSR1–6; Ig-Fn-Fn-Ig-Fn-Fn-Fn) and 11 C-zone super-repeats of 11 domains (CSR1–11, Ig-Fn-Fn-Ig-Fn-Fn-Fn-Ig-Fn-Fn-Fn) followed by 7 more domains. We consider the 183 domains from the first (A/I1) to the last Fn domain (A170) to be approximately associated with the myosin cross-bridge region of the filament (Figure 1). The bare zone segment comprising the last 10 Ig domains together with extensive inserted sequences is not shown.

The predicted ~ 4 nm span of each Fn and Ig domain leads to a C-zone super-repeat length of ~ 44 nm, strongly suggesting that the super-repeats are related to the accessory protein array [16]. Support for this idea comes from the observation of MyBP-C binding to the first Ig domain of each of those super-repeats [28]. MyBP-C is a multi-domain protein with 10 (skeletal) or 11 (cardiac) Ig and Fn domains [7]. Its three C-terminal domains (C8–10) which bind titin also bind strongly to the myosin tail [29,30] and are thought to run axially along the thick filament, whereas the N-terminal portion of the molecule is more transversely oriented and can interact with both myosin heads and the thin filament [7,31]. MyBP-C has an important role in modifying the acto–myosin interaction during muscle contraction [32,33].

The model of MyBP-C association with titin has been revised by data from two recent papers. When the molecular ruler hypothesis was tested by deletion of the first two titin C-zone super-repeats in a mouse model, the thick filaments became shorter by the expected amount (~ 4 × 43 nm = ~ 170 nm) [26]. Although it was also expected that the two MyBP-C stripes distal from the M-band would be ablated in the deletion mutant, only a single distal stripe was lost, leading to the conclusion that the first super-repeat is not involved in anchoring MyBP-C. In a more recent study, it was found that the 9 MyBP-C stripes correspond to the interface between the titin super-repeats from CSR2–3 to CSR10–11 [25]. In considering the implications of this result, it becomes clear that it is necessary to know the precise axial placement of titin along the whole thick filament.

One method to localise titin is to use antibodies against known epitopes to label muscle. If the domains are equally spaced, plotting the position of antibodies against the epitope domain number should give a linear relationship. The titin domain sequences in the cross-bridge region shows a strong uniformity in size: Ig domains are ~ 95 residues long and the Fn domains are ~ 100 residues in length, and domains in the same relative position in the C-zone super-repeats are highly conserved [34] with differences of only a few residues in size between them [35]. Almost all the differences are associated with intra-domain loops which would not affect the span of the domain. This uniformity between the domains suggests they are regularly spaced along the filament, a view reinforced by the structural analysis of two or three sequential domains which shows that they are closely associated linearly [[35], [36], [37]].

There is published antibody location data for A-band titin epitopes, particularly at the ends of the thick filament [17,22,23] and from near the M-line [27] (Figure 1 and Table 1). However, there has been a paucity of such data from the central cross-bridge region of the filament where MyBP-C is found. Raising antibodies is complicated by the high conservation of the repetitive sequences in this region, which leads to multiple binding sites for many titin antibodies [10,21]. To increase the coverage of the data, we have augmented the plot with data from several sources: we have characterised a *de novo* antibody, reinvestigated the binding domains and labelling positions of some of the antibodies used in early sequencing studies and, finally, determined the domains containing the epitopes for some antibodies which label multiple sites. Epitopes have been identified using recombinant titin fragments and Western blotting.

**Table 1:** Published titin antibody details

Antibody	Domain no/range (no. in Figure 4)	Spacing from M1 line (nm)	Method of analysis	Animal antibody raised in	Calibration	References
I41	A/I1 (− 13)	805	IEM	Rabbit	1.5 μm gap	[22]
I103	I102–104 (− 15)	820	SIM	Rabbit	direct LM	[17]
Ti-102	A/I7 (− 7)	780	IEM	Mouse	EM mag	[23]/[24]
MIR	A/I5–7 (− 8)	777	IEM	Human	1.5 μm gap	[24] [22]
A4	A4 (4)	735	IEM	Rabbit	1.5 μm gap	[22]
BD6	A9–21 (12)	718	IEM	Mouse	EM mag	[10]
A40–41	A40–41 (40.5)	600	IEM	Rat	MyBP-C = 43 nm	[25]
A77/78	A77 to A78 (77.5)	446	IEM	Rabbit	IEM allow for shrinkage	[26]
A80–82	A80-A82 (81)	430	IEM	Rabbit	MyBP-C = 43 nm	[25]
A165	A165 (165)	106	SIM	Guinea pig	direct LM	[26]

The antibody labelling positions in skeletal muscle rabbit and mouse myofibrils have been determined by immunofluorescence. Until recently, the positions of epitopes spaced less than 200 nm apart could only be determined by immuno-electron microscopy. However, the development of super-resolution light microscopy has enabled at least a 5-fold increase in resolution [38]. The advantage of these techniques is that structural changes and shrinkage from embedding and sectioning for electron microscopy are avoided. We have used two of these techniques, STORM (STochastical Optical Reconstruction Microscopy) and STED (STimulated Emission Deletion microscopy) [38], to investigate the position of antibodies to known domains of titin in the striated muscle thick filament.

We set out to establish the axial disposition of titin on the thick filament using epitope mapping and localising titin antibodies on myofibrils using super-resolution microscopy. Our data enables us to determine the titin locations associated with positions of the accessory protein stripes to within a few domains. Our results allow us to propose a correlation between the titin disposition and the arrangement of the myosin heads on the filament, lending further support for the role of titin as a blueprint for the thick filament.

## Results

### Titin antibodies and immunofluorescence in the cross-bridge region

**Figure 2:**
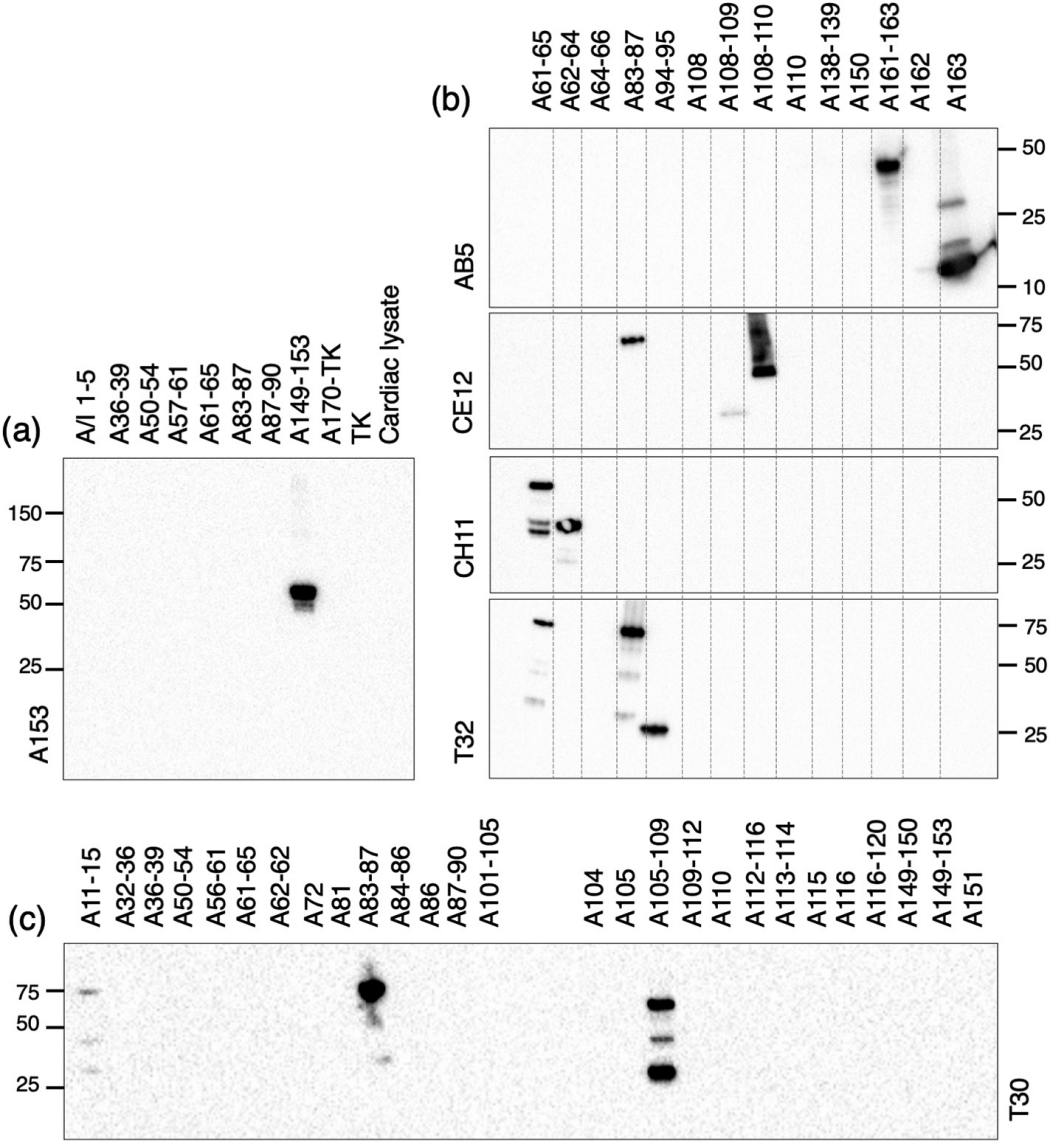
(a)–(c) Western blots of titin fragments probed with titin antibodies. (a) Antibody A153 detects only the fragment containing titin domain A153. (b) Of the four antibodies indicated AB5 and CH11 detect only one fragment, A163 and A62–64, respectively. CE11 and T32 each detect two fragments separated by 2 and 1 C-zone super-repeats. (c) T30 also recognises two sequences, A105–A106 and A83–A84, as well as weakly labelling A11–15 (see Supplementary data Figures 1, 3 and 4 for more detail).

**Figure 3:**
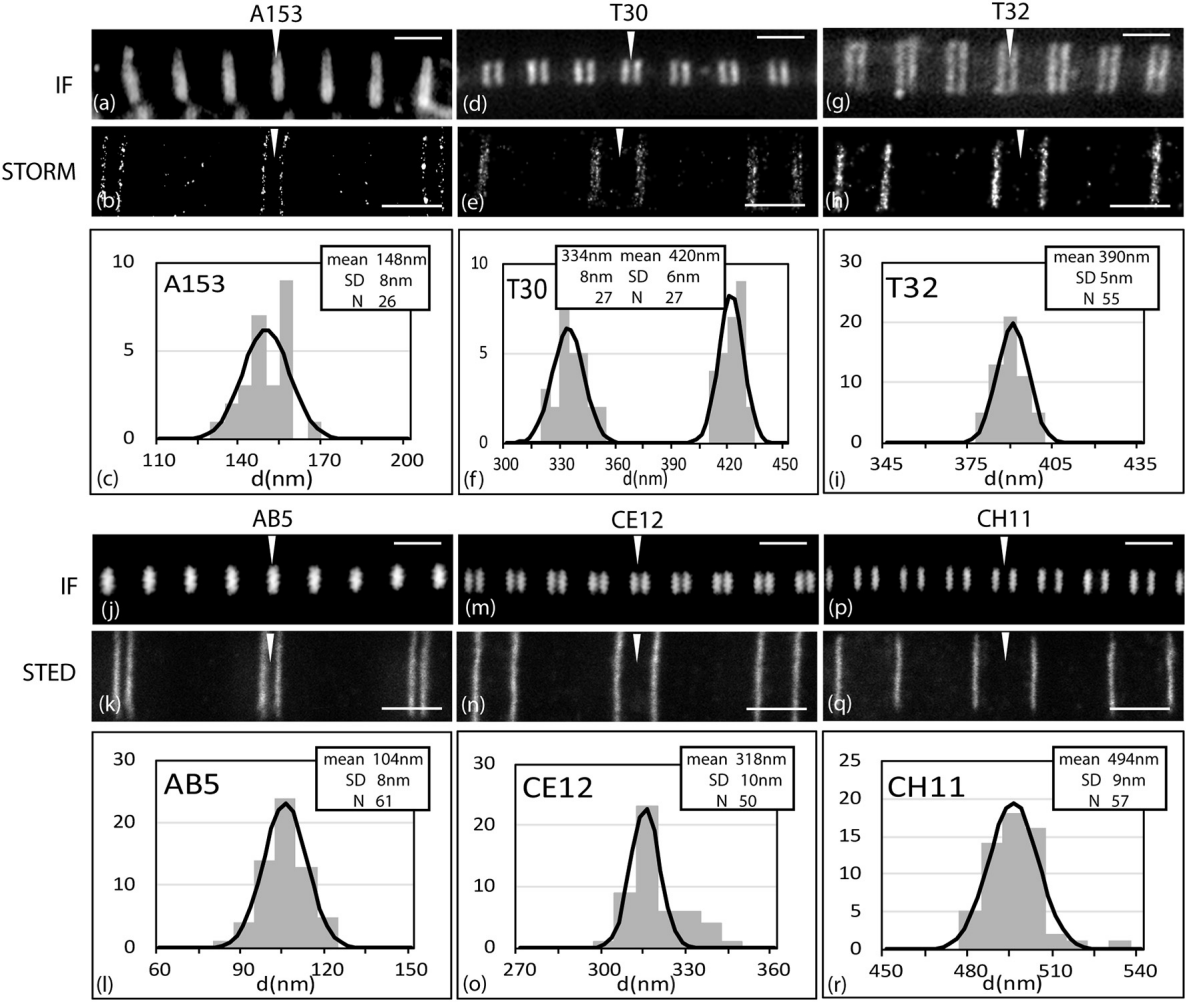
Rabbit psoas myofibrils labelled with titin antibodies and viewed in a standard microscope (IF) (a, d., g, j, m, p) and by STORM (b, e, h) or STED (k, n, q). Histograms and Gaussian fits of half doublet spacings giving distance from the centre of the thick filament (nm) (c, f, i, l, o, r). (a–c) A153; (d–f) T30; (g–i) T32; (j–l) AB5; (m–o) CE12; (p–r) CH11. Arrows indicate the position of the M-band. Note that T30 antibody (e) shows a closely spaced doublet in each half of the A-band. Scale bars IF images 2 mm, STORM and STED images 1 mm.

Several titin antibodies have been raised or recharacterized to locate known epitopes in the cross-bridge region of the muscle thick filament. The new antibody, A153, is a rabbit polyclonal raised against titin Ig domain A153, the first domain of CSR11 (red highlight Figure 1, Table 2). The antibody recognises uniquely a sequence containing A153 by Western blotting when compared to other sequences of titin including several that contain the equivalent domain from other C-zone super-repeats (Figure 2(a) and Supplementary Figure 1). By standard immuno-fluorescence the A153 antibody labels a narrow doublet across the M-band in rabbit myofibrils (Figure 3(a)). STORM and STED images locate the epitope to a narrow band ~ 150 nm from the centre of the M-band (Figure 3(b) and Supplementary Figure 2), specifically at 148 nm, SD ± 8 nm, n = 26 by STORM and 142 nm, SD ± 14 nm, n = 193 in STED (Table 2, Figure 3c and Supplementary Figure 2). Essentially the same results were obtained from mouse myofibrils. Although STORM imaging has a better resolution in principle, STED imaging is much faster and gave essentially the same result. Therefore, STED imaging was used where the highest resolution was not needed.

**Table 2:** Titin antibody details established in this study in rabbit psoas fibrils

Antibody	Domain no./range (no in Figure 4)	Spacing from M1 line (nm), SD, N	Method of analysis	Previous data	References, clone GenBank accession number
A153	A153 (153)	148 ± 8 nm, 26	STORM STED	None	
CH11	A62–63 (63)	494 ± 9 nm, 57	STED	Clone A58–69 EM 496 nm	[10,19,20], clone **X64698**, **X17329**
CE12	A109 (109)	318 ± 10 nm, 50	STED	EM 330 nm	[10]
	A87	–		Clone A86–A87	[19,20], clone **X64696** also **64699** and **64670**
AB5	A163 (163)	104 ± 8 nm, 61	STED	Clone A146–A174 EM 110 nm	[10,20], clone **X64697**
T30	A83–84 (83.5) A105–106 (105.5)	420 ± 6 nm, 27 334 ± 8 nm, 27	STORM	5 positions in chicken	[21]
T32	A83–84 (83.5) A94–95 (94.5)	390 + 21.5 nm 390–21.5nm^a^	STORM	Two positions in chicken	[21]

a Only one position seen but resolved into two 43 nm apart

A group of antibodies (BD6, CH11, CE12 and AB5) for which there are published data (Figure 1) are those that were raised, localised and used to isolate titin clones at early stages of sequencing the protein (Table 1, Table 2) [10,19,20]. The published clones associated with these antibodies are large, so it is not always clear what the precise epitope is. The CE12 clone T1 contained domains corresponding to A86–87, although there is some uncertainty about the longer clone sequence in the literature (initial data [19] cf. EMBL accession number **X64696** [20]). We re-examined the locations of the antibodies AB5, CE12 and CH11 (highlighted in green in Figure 1) and refined the epitope sequence they recognised (Figure 2, Figure 3, Table 2, Supplementary Figure 3).

STED microscopy of the AB5, CE12 and CH11 antibodies labelling rabbit myofibrils (Figure 3(k), (n) and (q)) locates them at 104 nm (SD 8 nm, n = 61), 318 nm (SD 10 nm, n = 50) and 494 nm (SD 9 nm, n = 57), respectively, from the centre of the thick filament, very close to the original EM values of 110, 330 and 496 nm [10] (Table 2, Figure 3(l), (o) and (r)). Human sequences encompassing the published and predicted domains were expressed and tested against the antibodies by Western blotting (Figure 2(b) and Supplementary Figure 3). The AB5 epitope was identified on A163 and CH11 recognised protein constructs spanning A62–63, both in agreement with the published clone data. CE12 recognised A87, as predicted, but also strongly recognised A109, the Ig domain in the same super-repeat position two repeats downstream. Interestingly, the extended upstream (RACE) sequence of the original clone in Labeit *et al*. [19] corresponds to that upstream of A109, suggesting that the sequences may be very similar. Indeed, in a comparison of all A-band domains A109 and A87 are two of the most closely related to each other with an identity of 65%. However, in native rabbit and mouse myofibrils CE12 binds only to one site.

As a further test of the alignment of titin, we have investigated two antibodies (shown in blue highlight in Figure 1) which in chicken muscle labels repetitive epitopes and which colocalise with MyBP-C antibodies to the resolution of EM [21]. The antibody T32 in chicken labels two stripes corresponding to AP#8 and #9 [21]. In STORM microscopy, it appeared to label only one site in rabbit and mouse myofibrils at ~ 380 nm from the centre of the filament (Figure 3(h) and (i), Table 2). This position lies between that expected for the two seen in chicken. This is consistent with the antibody labelling both, but we were unable to resolve two stripes separated by only 43 nm. We predicted that the binding for T32 to be close to A83–84 and A94–95 (see Materials and Methods); Western blots against expressed titin domains showed that this antibody did indeed label constructs encompassing the predicted binding sites, (Figure 2(b) and Supplementary Figure 3). The antibody also weakly recognises a construct including A61, two C-zone super-repeats upstream from A83 that is not detected by immunofluorescence.

Another antibody, T30, labelled at the position of 5 MyBP-C stripes (AP#6–#10) in chicken [21]. We have found, using STORM, that in rabbit it labels two closely spaced stripes in each half sarcomere (Figure 3(e)). The two are separated by 86 nm at positions 334 nm (SD ± 8 nm, n = 27) and 420 nm (SD ± 6 nm, n = 27) from the centre of the filament and closely correspond to MyBP-C stripes #5 and #7 (AP#7 and #9) (Figure 3(f), Table 2). Western blotting of expressed human titin fragments located the antibody binding site to a region linking the ends of A83 and A105 to their following domains (Figure 2(c) and Supplementary Figure 4). Alignment of the sequence of C-zone super-repeat domains 8 and 9 identified an identical 9-residue sequence at the 8 to 9 domain boundaries in the five titin regions predicted to bind T30 in chicken (Supplementary Figure 5). This sequence is conserved in four of the five same regions in rabbit and human, and the two of the corresponding domains tested are recognised in blots, whilst the diverged sequence linking A116–117 was not (Figure 2(c)). It is unclear as to why only two of these positions were decorated in STORM experiments. A partially homologous sequence linking A11–12 was also recognised weakly by T30 in blots (Figure 2(c)).

### Regular arrangement of titin

**Figure 4:**
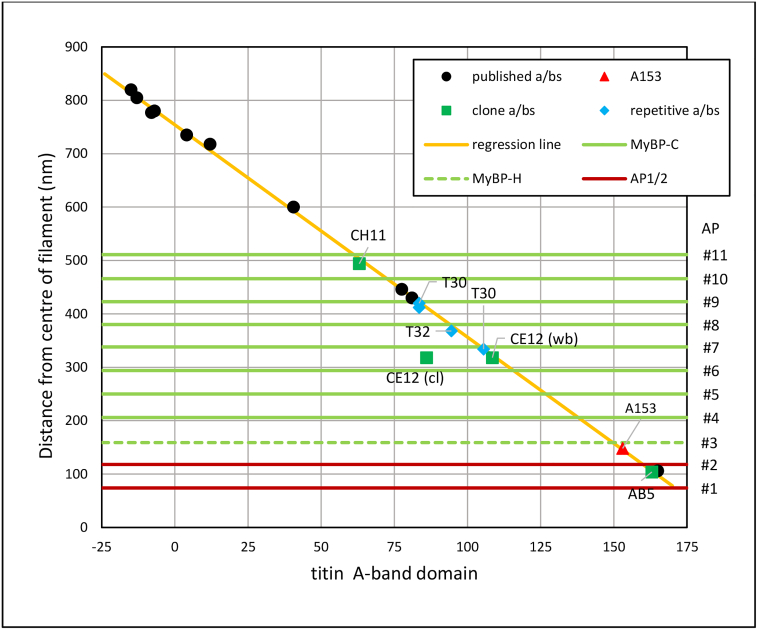
Positions of titin antibodies with respect to the centre of the filament plotted against the number of the relevant titin domain. The 14 A/I domains are numbered − 13 to 0 and domains 1–170 correspond to A1–A170 (Figure 1 and Table 1, Table 2). Black circles; published data (unhighlighted antibodies in Figure 1 and details in Table 1). Coloured symbols are labelled and correspond to antibodies that have been newly or further analysed here (Figure 1 and Table 2). Red triangle; new antibody A153. Green squares; “clone” antibodies reassessed. Blue diamonds; repetitive antibodies. The regression line shown was calculated in Excel from all the data except for the outlier, CE12 (cl). The numbers #1–#11 show the positions of the 11 accessory proteins. The green horizontal lines show the observed position of the eight MyBP-C stripes (#4–#11) and (dotted) the MyBP-H stripe (#3) in the A-band of rabbit psoas muscle [5]. The two brown lines indicate the positions of the first two accessory protein stripes (AP#1–#2) of unknown provenance nearer the bare zone.

The plot of antibody positions (distance from the centre of the M-band) against the domain number of the titin epitope shows a linear relationship as expected (Table 1, Table 2, Figure 4). All of the points fall close to a straight line except for the outlier CE12(Cl), indicating that the additional epitope identified in Western blots (CE12(wb)), A109, is that labelled in rabbit myofibrils rather than the domain, A87, identified by the antibody in the initial cloning of titin. The regression line calculated from all the data except the outlying CE12 data point is linear according to the Wald–Wolfowitz runs test and the data points fit the line with an *R*^2^ = 0.999 (Table 3). The slope of the regression line is − 3.98 nm/domain and gives the average span of each domain along the filament axis. This corresponds very well to the size of Ig and Fn domains in the literature where lengths between 4 and 4.5 nm are found [39]. The *y* intercept at *x* = 0 corresponding to domain A/I14 is 754 nm. These regression line characteristics position the first Fn domain (Fn1, A/I2, in Figure 1) at 806 nm from the centre of the M-band in agreement with this domain being at the end of the thick filament of length 1.6 mm. The final Fn domain, A170, is predicted to locate to 78 nm from the centre of the filament, close to the edge of the bare zone of width 154 nm [9]. The residuals of the data points from the line are on average 4 nm with a maximum of 12 nm; that is, the points deviate from the regression line on average by less than the span of two domains.

**Table 3:** Linear regression line calculations for titin antibody data plotted in Figure 4

Domain range (number in Figure 4)	*R*^2^	Slope (nm/domain) (95% confidence limits)	Intercept at *x* = 0 (A/I14) (95% confidence limits)
All A/I1–A170 (− 13 to 170)	0.999	− 3.98 (− 3.92 to − 4.03)	754 nm (749–758 nm)
D-zone A/I1–A61 (− 13 to 61)	0.995	− 4.02 (− 3.75 to − 4.28)	754 (747–762 nm)
C-zone A40–A165 (40–165)	0.999	− 3.89 (− 3.81 to − 3.96)	742 (734–751 nm)

Thus, we have strong evidence that titin is linearly and uniformly extended along the whole of the cross-bridge region of the thick filament. From this fit, we are able to associate the features of the filament with specific regions of titin to within a few domains. In particular, the C-zone super-repeat of 11 domains extends ~ 11 × 4 = 44 nm, very close and within the error to both the MyBP-C periodicity and the myosin helical repeat, lending further support to the idea of an intimate relationship between the three proteins.

### Relationship of titin to MyBP-C positions

**Figure 5:**
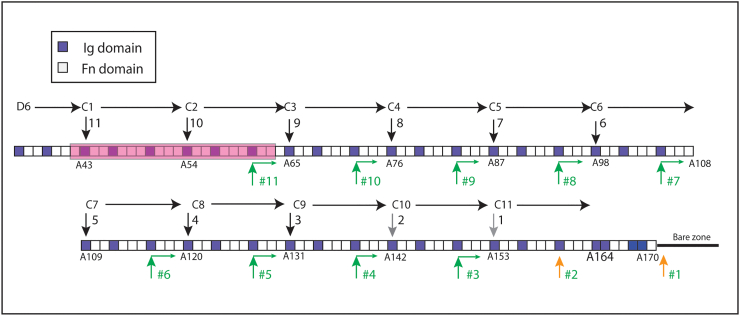
Diagram showing the relationship of C-zone titin domains to MyBP-C and accessory protein stripes. The region A42–A63 coloured in red is the region of titin deleted by Tonino *et al*., which covers CSR1 and most of the CSR2 [26]. The black vertical arrows indicate the first domain in the 11 C-zone super-repeats and the positions previously assumed to be the MyBP-C binding sites. The green vertical arrows show the positions determined in this study for the nine MyBP-C stripes. The horizontal component indicates the axial disposition of the C terminal domains (C8–10) of MyBP-C molecules towards the centre of the filament. The accessory protein stripe number (#1–#11) is given against the green and orange arrows. Stripes #1 and #2 are of unknown origin.

Of the 11 accessory protein stripes separated by ~ 43 nm seen in each half of the thick filament, MyBP-C is found on the most distal 7–9, depending on the muscle type [5,6,[40], [41], [42]]. Their positions as determined by immuno-electron microscopy in fast and slow skeletal and cardiac muscle are very similar [5,25,31,42]. Horizontal lines showing the position of these stripes in relation to the regression line are shown in Figure 4 (data for rabbit psoas muscle taken from Bennett *et al.* [5]). The position of two of the titin antibodies, CH11 and A153 at 494 and 148 nm, respectively, correspond closely to the spacing of the first and last of the 9 MyBP stripes at ~ 160 and ~ 500 nm. We can therefore define the region of titin associated with MyBP-C to be between the two corresponding epitopes, that is, from ~ A60 to ~ A153. We can determine more specifically the titin domains corresponding to the MyBP-C stripes from their position with respect to the regression line (Table 4). Using the data for the positions of the eight MyBP-C stripes in rabbit psoas muscle [5], the equivalent titin domains start at A61 and finish at A138, spanning 77 domains. This is equivalent to 11 domains per stripe, direct evidence in support of the idea that MyBP-C is associated with the 11-domain super-repeat of titin. Given the spacing per domain of 3.98 nm, this equates to a 43.8-nm stripe separation. Of particular interest is the observation that the 11 accessory protein stripes do not directly correlate with the 11 C-zone super-repeats of titin; the most distal MyBP-C position (Stripe AP #11) is not found at the beginning of the first super-repeat (A43–A53) but locates almost two super-repeats away towards the end of the CSR2 (compare black and green arrows in Figure 5). This result agrees with a previous analysis which used three titin antibody locations near the MyBP-C zone [25].

**Table 4:** Determination of titin domain corresponding to MyBP-C positions using regression line data from Figure 2 (slope − 3.98 nm/domain, intersection 754 nm)

	Bennett *et al*. [5], rabbit psoas sections			Tonino *et al*. [25], mouse heart sections			Lee *et al*. [31], mouse heart fibrils		
AP/MyBP-C position	MYBP-C data nm	Titin domain	CSR-dom	MyBP-C data nm	Titin domain	CSR-dom	MyBP-C data nm	Titin domain	CSR-dom
AP#11/C#9	511	61.1	**2–8**	505	62.6	**2–10**	501.2	63.6	**2–11**
AP#10/C#8	466	72.4	**3–8**	461	73.7	**3–10**	458.3	74.4	**3–10**
AP#9/C#7	423	83.2	**4–8**	419	84.3	**4–10**	415.4	85.2	**4–10**
AP#8/C#6	380	94.1	**5–8**	375	95.3	**5–9**	372.2	96.0	**5–10**
AP#7/C#5	338	104.6	**6–8**	332	106.1	**6–9**	329.4	106.8	**6–10**
AP#6/C#4	294	115.7	**7–8**	289	117.0	**7–9**	286.1	117.7	**7–10**
AP#5/C#3	250	126.8	**8–8**	247	127.5	**8–9**	243.1	128.5	**8–10**
AP#4/C#2	206	137.8	**9–8**	203	138.6	**9–9**	200	139.3	**9–9**
AP#3/C#1 H	(162)^a^	(148.8)^a^	**(10-8)**^a^	162	148.9	**10-8**	157.2	150.1	**10–9**
	159	149.6	**10–9**						
AP#2	119^b^	159.8							
AP#1	75^b^	170.7							
domains/repeat		10.96			10.78			10.81	

The numbers are in bold to accentuate them and make them easily comparable across the table.

a Inferred data.

b Calculated value assuming 43.6 nm stripe separation relative to MyBP-C stripe #2.

There is less certainty about the precise position of the MyBP-C with respect to the titin C-zone super-repeats. The titin domain identified as the centre of the MyBP-C stripe using the rabbit data (Figure 4) is the eighth domain of each super-repeat (Table 4). A previous study using mouse heart identified domain 10 as the site of the stripes [25]. Using the titin regression line data for two published sets of MyBP-C data from mouse heart, both using the same antibody, the titin domain identified varies according to the super-repeat from domain 8 to domain 11 (Table 4) [25,31]. Whilst this difference of a few domains is within the errors of the experiments, one of the influencing factors is the calibration used for the MyBP-C data. One method [5] used the distance between the cross-bridge gaps at the ends of the filament, previously determined to have a spacing of 1.5 μm [9]. This gives a MyBP-C spacing of 43.6 nm, close to 11 times the domain spacing in the titin data (43.8 nm). Both other sets of data are based on a 43-nm repeat for MyBP-C leading to a shift over 9 repeats of 8 × 0.8 nm = 6.4 nm (1–2 domains) away from the titin repeat. The least difference between the three sets of data is for the smaller separation stripes AP #3 and #4 (MyBP-C #1 and #2) giving domain 8 or 9 for all three sets of data. Since calibration factors will have the smallest effect on small spacings, it seems that this is the most likely location for the MyBP-C stripes with respect to the titin C-zone super-repeat.

Using antibodies specific to MyBP-C domains showed that domains C0-C7 have similar axial positions [31], in support of the idea that most of the molecule lies transverse to the filament axis to form the visible stripes in unlabelled muscle and isolated A-bands (A-segments). The C-terminal part of the molecule, homologous to MyBP-H, extends along the filament towards the M-line by 5–10 nm [31]. MyBP-C may, therefore, have an extended binding site with titin. If, as we suggest, the body of the MyBP-C stripe is at domain 8 in the C-zone super-repeat, the binding site would extend ~ 2 domains C-terminal from this to super-repeat domain 10 (Figure 5).

The position of 9 MyBP-C stripes at the end of CSR 2–10 leaves the first two accessory protein stripes close to the bare zone to be accommodated. Assuming the same spacing as MyBP-C and the first MyBP-C stripe associated with A149 (domain 8 of the CSR10), these two extra stripes would fall close to A160 and just beyond A170, the last Fn domain (Figure 5, Table 4). There is no information as to what composes these stripes, but the site for stripe 2 towards the end of CSR11 is similar in domain organisation to MyBP-C locations whilst the stripe 1 location is not.

### Titin and myosin organisation

**Figure 6:**
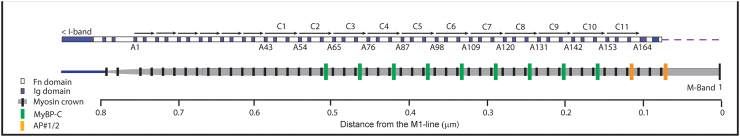
Diagram showing the alignment of A-band titin with respect to the cross-bridge region of the thick filament as determined by the present data. Top. A model of A-band titin showing Fn (light) and Ig (dark) domains. Arrows (unlabelled) show the D-zone super repeats and (labelled) the C-zone super-repeats. Bottom. Model of the thick filament on the same axial scale as titin. The arrangement of 49 myosin crowns along one half of the thick filament is shown by the black lines. The positions of the 9 MyBP-C stripes are shown in green and the other two accessory protein stripes in orange (data taken from Craig and Megerman [1]). Scale shows the distance from the centre of the thick filament.

We can estimate the position of each of the 49 levels of myosin heads in each half of the muscle thick filament [9] in relation to the titin domain structure using the suggestion that the heads associate with the groups of 2 or 3 Fn domains in the titin sequence (Figure 6) [43]. This is consistent with three myosin levels in a C-zone super repeat and two in a D-zone super repeat. Studies of A-segments and cryosections have located the myosin cross-bridge positions with respect to the accessory protein stripes [1,3] and show the first stripe at the edge of the bare zone just before the first cross-bridge level. With three levels between each stripe, the seventh crown level is just after AP#3, the first MyBP-C stripe at 162 nm (A149) (Figure 5). We therefore assign the seventh cross-bridge layer to the 3 Fn subgroup A146–148 at ~ 168 nm [25]. Of the first six myosin positions between here and the bare zone, four can be associated with Ig2Fn or Ig3Fn subgroups up to the end of CSR11. The pattern of the last seven titin domains up to A170, Ig,Ig,Fn,Fn,Ig,Ig,Fn, has a Fn doublet at A166 and A167, but the last three domains (A168–170) have little obvious relationship to other A-band domains and an unknown relationship to myosin heads (see Supplementary Table 2).

From the proposed seventh myosin layer position at A146–148 to the beginning of the first D-zone super-repeat near the tip of the filament, there are 40 Ig2Fn and Ig3Fn subgroups, which would locate crown 47 at A1–3 (Figure 1, Figure 6). The cross-bridge gap next to crown 47 at the end of the filament would therefore correlate with the subsequent titin sequence, the run of 6 Fn domains A/I 9–14. A/I14 is the domain that corresponds to the calculated intersection of the regression line at 754 nm in Figure 4. This is almost exactly the measured gap position of 750 nm [9]. The final two levels of heads would fall at the start of the group of 6Fn domains and A/I6–7, in line with previous immuno-EM results [22].

To maintain a constant myosin axial repeat along the filament, the average titin domain separation in the D-zone will be (2 × 11)/(3 × 7) = 22/21 times that of the C-zone; that is, ~ 5% longer (~ 0.2 nm). To assess whether this is the case, the titin antibody data were separated into two overlapping groups, A/I1-A61 (D-zone) and A40-A165 (C-zone), and the regression line for the two regions calculated (Table 3). The resulting slope for the D-zone was − 4.02 nm/domain and for the C zone − 3.89 nm/domain, giving a possible small increase in domain spacing of ~ 3% in the D-zone supportive of this idea. However, the values are within the 95% confidence limits of each other and require further data to assess their significance.

## Discussion

The underlying structure of the muscle thick filament is determined by the strong interactions of myosin itself leading to the intrinsic ~ 14.5 nm repeat. Titin modulates the structure, coercing myosin into a filament of a well-defined length. Using characterised antibodies together with published data, we have been able to show that titin is linearly and uniformly arranged along the cross-bridge region of the thick filament. Furthermore, it is the Fn-rich sequence of titin that spans the length of this region. As near as can be determined the MyBP-C repeat and that of the titin C-zone super-repeat sequence are the same, at 43-44 nm, and MyBP-C is associated with CSR2–10 as recently suggested [25].

### Titin-MyBP-C alignment

The apparent identity of the myosin helical repeat, the MyBP-C stripe separation and the titin 11-domain super-repeat raises the question of what interactions determine the structure of the thick filament. *In vitro*, MyBP-C binds more strongly to myosin than titin and binds at a 1:1 ratio to the myosin tail [29,44]. At this high concentration, it interferes with the formation of well-ordered myosin filaments, giving rise to thinner, poorly ordered structures [29]. However, in the sarcomere, it is present only in the C-zone at a third of this concentration and is limited to a set of well-defined positions. It is very likely that it is the disposition of titin on the thick filament that sets this limit. It is possible that the six molecules of titin associated with each half thick filament [45] block the myosin binding sites for MyBP-C in two axial positions out of three in the C-zone and all sites elsewhere. The principal binding site of MyBP-C for myosin has been identified as the C-terminal Ig domain [30]. However, three or four C terminal domains have been found necessary for strong localisation to the A-band in transfected chick myoblasts [46], whilst the last domains were also found to bind recombinant titin fragments *in vitro* [28]. To accommodate three or four MyBP-C domains raises the possibility that an extensive binding site on titin is required in addition to that on myosin.

*In vitro* evidence showed that essentially all 11 of the first titin Ig domains in the C-zone super-repeats could bind MyBP-C in dot-blots [28]. It is now clear that the 9 MyBP-C stripes are not located near the first two of these Ig domains. Further, the binding site for MyBP-C identified here corresponding to titin C-zone super-repeat domains 8 to 10, puts into question the role of the first Ig domain in MyBP-C binding, at least as the sole binding site. In support of this, the deletion of the first 2 C-zone super-repeats resulted in the loss of only the most distal MyBP-C stripe [26]. The exons deleted, 305–325, correspond to domains A42–A63; that is, one domain N-terminal to the normally defined CSR1 and CSR2 domains (A43–A64) [16] (Figure 5). This is consistent with the loss of the first MyBP-C binding site that we identify near the end of CSR2, corresponding to A61–63, but leaves two of the putative binding domains, Fn11 and Ig1, and could explain the “ghost” of the stripe sometimes seen in this earlier work [25].

Are there features within titin that would explain the lack of binding of MyBP-C to CSR1 as well as to CSR11? Interestingly, in a Clustal alignment analysis of titin domains, Fn domain 10 of CSR1 (A52) was more similar to domain 6 of D6 super-repeat (A41) than to Fn 10 of CSR2–10 [34] (Supplementary Table 2). It seems likely that these differences will give rise to a conformation that is inimical to MyBP-C binding. Further, a comparison of the sequences of seven domains across the interface of C-zone super-repeats from domain 8 in one to domain 3 in the next found that this sequence over the beginning of CSR1 and 2 was less similar to that for MyBP-C binding regions [25]. This was particularly evident in the distribution of well-conserved surface residues. Perhaps significantly, many of these differences were in domains 8 and 9, near where we place MyBP-C. The availability of more sensitive and quantitative binding-assays like isothermal titration calorimetry nearly 25 years after the work by Freiburg and Gautel [28] should now allow quantitative analysis of the interactions between MyBP-C, titin and myosin.

### Titin and myosin organisation

There is little detail concerning the binding of titin to myosin. The myosin tail has been shown to bind to titin in dot blots and solid-phase assays and has been visualised bound to titin under the electron microscope [20,47,48]. In addition, it has been shown that the groups of 2 or 3 Fn domains can bind to myosin subfragment 1 and, in addition, can affect the Ca^2+^ sensitivity of force development in cardiomyocytes [43]. We have shown that the organisation of myosin in the thick filament in relation to titin is compatible with this suggestion and that essentially all the Fn groups could be locations for myosin heads (Figure 6). However, the deletion of the 14 A/I domains at the tip of the filament in an earlier attempt to demonstrate the role of titin in maintaining filament length did not change the length of the filament [17]. Most of these domains are in the vicinity of heavy meromyosin and cannot interact with the part of the myosin tail that has been shown to interact most strongly with titin [22,47,49]. This suggests that the titin–myosin tail interaction is the stronger in determining the length and structure of the thick filament. Titin's interaction with myosin heads may have a secondary, albeit important, functional role.

The association of myosin heads with the groups of two or three titin Fn domains may locally influence the arrangement of the three layers of myosin within a C-zone super-repeat. Considering the Fn domains whose surface features are most highly conserved, Muhle-Goll *et al*. [43] suggest that domains 2, 6 and 9, will be the domains strongly involved. Such a relationship may distort the myosin heads (and tails) from a simple helical disposition, thus giving rise to the 43-nm pseudo-helical myosin repeat in relaxed muscle observed by X-ray diffraction [50] and the axial and azimuthal distortions of myosin heads from an ideal helix seen in EM reconstructions of thick filaments [51,52]. The reconstructions also show a short length of three filament-bound domains, which is attributed to MyBP-C (presumably C8-C10), which may also contribute to myosin head disposition.

In the D-zone where two layers of myosin will be associated with a super-repeat, it has been observed that in rabbit myofibrils some of the cross-bridge levels at the end of the A-band are paired [4], suggesting that here, also, the cross-bridge spacing may be modulated by the underlying pattern of titin domains.

The axial alignment of titin with the thick filament (Figure 6) reveals how many of the structural features of the thick filament can be understood in terms of titin being a molecular ruler [[10], [11], [12]]. However, how these interactions between myosin, MyBP-C and titin are achieved and relate to their functional importance is still poorly understood. Furthermore, active contraction, lower temperatures and stretching titin by extending relaxed muscle all lead to an increase in the myosin head repeat and an unknown mechanosensitive change in the relationship of myosin with titin and MyBP-C [50,53,54].

### Mutations of Titin and MyBP-C and myopathies

Mutations in titin, MyBP-C and myosin give rise to many forms of heart disease and between them appear to be associated with a majority of HCM cases [13,55]. Many mutations affect the folding and stability of MyBP-C or titin domains, but changes to surface residues at interfaces may also affect the interaction of titin, MyBP-C and myosin. The data we present here reveals a clearer picture of where these interactions will occur and will allow a better focus on vulnerable interaction sites. The Fn domain A150, for example, is a hot spot for mutations that lead to hereditary myopathy with early respiratory failure (HMERF) [56,57] and an increased risk for cardiac conduction abnormalities [58]. On the present analysis, titin A150 is at the level of the first MyBP-C stripe, an observation that could be useful when interrogating the molecular pathology of the disease.

## Materials and Methods

### Myofibril preparation

Myofibrils were prepared from rabbit and mouse skeletal muscle. The animals were treated in accordance with EEC regulations. Bundles of glycerinated rabbit psoas were prepared as described in [59] and stored at − 20 °C. Myofibrils were prepared from bundles by standard methods [60]. Mouse myofibrils were prepared from EDL muscle essentially as previously described [61].

### Antibodies

Production of an antibody against titin A153 was carried out using recombinant A-band Ig-domain A153 (residues 32,007–32,105 of transcript NM_001267550.1). Briefly, the sequence encoding A153 was amplified by PCR from a human cDNA library, cloned into a modified pET vector with an N-terminal His6-tag and TEV cleavage site and verified by DNA sequencing. The protein was expressed, harvested and lysed as described below, purified by nickel affinity and gel filtration chromatography and used for immunisation of rabbits by a commercial supplier (BioScience bj-diagnostik GmbH, Göttingen, Germany) after cleavage of the tag by TEV protease. The specific immuno-reactivity to titin was verified by Western blot to cardiac and skeletal tissue samples. The specificity of the resulting antibody was shown by Western blots against expressed fragments of titin (Supplementary Figure 1).

Monoclonal antibodies CE12, CH11 and AB5, originally raised by Dr. John Trinick [10], were generously supplied by Dr. Michelle Peckham. They were used to recognise clones in the original sequencing studies of titin [10,19,20], and we therefore refer to them as “clone” antibodies. The published clones (see Table 2) are of variable length and do not always pinpoint the epitope. To narrow this down a preliminary regression line calculated from published positions of titin antibodies with known epitopes was generated. Using the spacing determined by immunofluorescence, the epitopes the antibodies recognise in rabbit were predicted and characterised more fully by Western blots against various recombinant fragments of titin. Titin antibodies T30 and T32, monoclonal mouse antibodies, which recognise repetitive sites in chicken breast muscle, were a gift from Dr. Dieter Fürst [21]. The epitopes they recognise were not known. They were determined as above for the “clone” antibodies.

### Protein expression and epitope characterisation

Recombinant titin fragments used for antibody epitope identification were recombinantly expressed in *Escherichia coli* RIPL (DE3) with an N-terminal His_6_-tag in LB media at 37 °C. Protein expression was induced for 3 h following addition of IPTG to 0.5 mM and cultures were harvested by centrifugation. Cells were lysed by incubation in BugBuster (Millipore), 4 mg/ml DNAse I (Sigma) and 1 mM MgCl_2_ for 15 min at room temperature, with samples then mixed with SDS-PAGE loading buffer.

Samples were run on SDS-PAGE (including a sample of rat cardiac tissue for A153 characterisation), transferred to nitrocellulose membrane and blocked with 5% w/v milk in low salt buffer. The membranes were then incubated with primary antibodies for 2–4 h at room temperature at dilutions of 1:100–1:1000, washed in low-salt buffer (10 mM Tris (pH 7.4), 150 mM NaCl, 1% v/v Triton X-100; 3 × 10 min washes), incubated in HRP-conjugated mouse or rabbit secondary antibody for 1 h at room temperature and then washed again in low-salt buffer (3 × 10 min washes). HRP activity was then visualised by incubation with ECL (GE healthcare) on a Bio-Rad imaging system.

### Immunofluorescence

Myofibrils were applied to slides or coverslip-bottomed dishes then fixed with 4% PFA for 5 min before blocking with 1% BSA in PBS. They were incubated in primary antibody in the same buffer for 1 h before washing and subsequent incubation in secondary antibody. For routine fluorescence, goat anti-rabbit IgG tagged with Cy2 (Jackson 111-225-144) and goat anti-mouse Cy3 (Jackson 115-165-146) were used. For STORM microscopy, goat secondary antibodies labelled with Alexa647 from Invitrogen were used (anti-rabbit A21244; anti-mouse A21244). For STED microscopy, secondary antibodies were labelled with STAR RED or STAR 580 (Abberior) or Atto647N (Sigma).

Standard immunofluorescence microscopy was carried out on a Zeiss Axiovert 200M.

For STORM, myofibrils were viewed in a Nikon Eclipse Ti-E inverted microscope equipped with an Andor iXon EMCCD camera in the Nikon Centre, Kings College London. Images were collected with Nikon software, NIS. The data were analysed either with the Nikon software or with the ThunderSTORM plugin in ImageJ [62]. Final images were saved at a pixel size of 10 nm. STORM microscopy locates individual blinking fluorophores from many short exposures and in principle can have a high resolution [63]. However, if the density of blinking fluorophores is too high, the analysis can underestimate the separation in a closely spaced doublet. This can be remedied using the plugin HAWK to ensure that the data was not affected or to remedy the problem if it was [64]. Our initial data were submitted to HAWK analysis but since there was no improvement in separation or resolution of the fluorescent doublets, it was determined that the data were not too dense, and this step was omitted.

STED microscopy uses structured illumination to produce images with around 30–50 nm resolution depending on the fluorophore used [65]. Best images were obtained using the fluorophore STAR RED or Atto647N. STED was carried out on a STEDYCON (Abberior) attached to a Leica TCS SP5 ll confocal microscope. Images were recorded at a pixel size of 15 nm. Both STED and STORM images were obtained for some antibodies and gave essentially the same results (see Supplementary Figure 2).

### Analysis

Analysis of the STORM and STED images was carried out in ImageJ. Images were oriented such that one or more doublets were vertical. A rectangular mask defining a well-ordered region was used to generate a profile of the doublets and the positions of peak intensity identified. Alternatively, the positions of the doublet lines were determined with a cursor by eye. Both methods gave essentially the same doublet separation. The spacing from the centre of the filament was obtained by dividing this distance by 2. The histogram of the data was generated and a Gaussian fit to the data obtained in Excel.

## Appendix A Supplementary data

Supplementary material Image 1
