# Supplementary material for: The Axial Alignment of Titin on the Muscle Thick Filament Supports Its Role as a Molecular Ruler
Source: J Mol Biol. 2020 Aug 7;432(17):4815–29. doi: 10.1016/j.jmb.2020.06.025 (PMC7427331; doi:10.1016/j.jmb.2020.06.025)
Supplement: Supplementary file 1 — Supplementary material [file mmc1.docx]

**Supplementary data**

**Identification of epitopes of T30 antibody.**

The titin antibody T30 has been shown to recognise repetitive epitopes in chicken breast muscle. We found that it recognised two sites in rabbit psoas muscle separated by 86 nm and lying close to the MyBP-C binding sites #5 and #7. We could predict that the epitopes would be close to domains A83 and A105, the eighth domain of the CSR5 and 7 respectively. Testing the antibody against a number of expressed titin fragments we could indeed show that the epitopes recognised were within the fragments between A83-A87 and A105-A109 (Supplementary Fig. 2). Using smaller fragments, the epitope recognised was the boundary between the Ig8 and Fn9 domain of the 5^th^ and 7^th^ C-zone super-repeat corresponding to A83/84 and A105/106. In the chicken where the antibody recognises 5 sites, nine residues of the boundary sequences are completely conserved (boxed in Supplementary Fig. 5a). The same sequence is present in human, mouse and rabbit in four of the five positions, the exception being A116-117. In Western blotting of expressed human Titin fragments T30 recognises the two tested fragments containing this sequence and does not recognise the sequence that is diverged from chicken in human, rabbit and mouse.

| A/I | A/I1 | A/I2 | A/I3 | A/I4 | A/I5 | A/I6 | A/I7 |  |  |  |  |
| --- | --- | --- | --- | --- | --- | --- | --- | --- | --- | --- | --- |
|  | 108 | 1 | 2 | [3](http://www.rcsb.org/pdb/explore/explore.do?structureId=4o00) | 109 | 4 | 5 |  |  |  |  |
|  | **A/I8** | **A/I9** | **A/I10** | **A/I11** | **A/I12** | **A/I13** | **A/I14** |  |  |  |  |
|  | 110 | 6 | 7 | 8 | 9 | 10 | 11 |  |  |  |  |
| D1 | **1** | **2** | **3** | **4** | **5** | **6** | **7** |  |  |  |  |
|  | 111 | 12 | 13 | 112 | 14 | 15 | 16 |  |  |  |  |
| D2 | **8** | **9** | **10** | **11** | **12** | **13** | **14** |  |  |  |  |
|  | 113 | 17 | 18 | 114 | 19 | 20 | 21 |  |  |  |  |
| D3 | **15** | **16** | **17** | **18** | **19** | **20** | **21** |  |  |  |  |
|  | 115 | 22 | 23 | 116 | 24 | 25 | 26 |  |  |  |  |
| D4 | **22** | **23** | **24** | **25** | **26** | **27** | **28** |  |  |  |  |
|  | 117 | 27 | 28 | 118 | 29 | 30 | 31 |  |  |  |  |
| D5 | **29** | **30** | **31** | **32** | **33** | **34** | **35** |  |  |  |  |
|  | 119 | 32 | 33 | 120 | 34 | 35 | 36 |  |  |  |  |
| D6 | **36** | **37** | **38** | **39** | **40** | **41** | **42** |  |  |  |  |
|  | 121 | 37 | 38 | 122 | 39 | 40 | 41 |  |  |  |  |
| C1 | **43** | **44** | **45** | **46** | **47** | **48** | **49** | **50** | **51** | **52** | **53** |
|  | 123 | 42 | 43 | 124 | 44 | 45 | 46 | 125 | 47 | 48 | 49 |
| C2 | **54** | **55** | **56** | **57** | **58** | **59** | **60** | **61** | **62** | **63** | **64** |
|  | 126 | 50 | 51 | 127 | 52 | 53 | 54 | 128 | 55 | 56 | 57 |
| C3 | **65** | **66** | **67** | **68** | **69** | **70** | **71** | **72** | **73** | **74** | **75** |
|  | 129 | 58 | 59 | 130 | 60 | 61 | [62](http://www.rcsb.org/pdb/explore/explore.do?structureId=1BPV) | 131 | 63 | 64 | 65 |
| C4 | **76** | **77** | **78** | **79** | **80** | **81** | **82** | **83** | **84** | **85** | **86** |
|  | 132 | [66](http://www.rcsb.org/pdb/explore/explore.do?structureId=3LPW) | [67](http://www.rcsb.org/pdb/explore/explore.do?structureId=3LPW) | 133 | 68 | 69 | 70 | 134 | 71 | 72 | 73 |
| C5 | **87** | **88** | **89** | **90** | **91** | **92** | **93** | **94** | **95** | **96** | **97** |
|  | 135 | 74 | 75 | 136 | 76 | 77 | 78 | 137 | 79 | 80 | 81 |
| C6 | **98** | **99** | **100** | **101** | **102** | **103** | **104** | **105** | **106** | **107** | **108** |
|  | 138 | 82 | 83 | 139 | 84 | 85 | 86 | 140 | 87 | 88 | 89 |
| C7 | **109** | **110** | **111** | **112** | **113** | **114** | **115** | **116** | **117** | **118** | **119** |
|  | 141 | 90 | 91 | 142 | 92 | 93 | 94 | 143 | 95 | 96 | 97 |
| C8 | **120** | **121** | **122** | **123** | **124** | **125** | **126** | **127** | **128** | **129** | **130** |
|  | 144 | 98 | 99 | 145 | 100 | 101 | 102 | 146 | 103 | 104 | 105 |
| C9 | **131** | **132** | **133** | **134** | **135** | **136** | **137** | **138** | **139** | **140** | **141** |
|  | 147 | 106 | 107 | 148 | 108 | 109 | 110 | 149 | 111 | 112 | 113 |
| C10 | **142** | **143** | **144** | **145** | **146** | **147** | **148** | **149** | **150** | **151** | **152** |
|  | 150 | 114 | 115 | 151 | 116 | 117 | 118 | 152 | 119 | 120 | 121 |
| C11 | **153** | **154** | **155** | **156** | **157** | **158** | **159** | **160** | **161** | **162** | **163** |
|  | 153 | 122 | 123 | 154 | 124 | 125 | 126 | 155 | 127 | 128 | 129 |
| P | **164** | **165** | **166** | **167** | **168** | **169** | **170** |  |  |  |  |
|  | 156 | 157 | 130 | 131 | 158 | 159 | 132 |  |  |  |  |

**Supplementary Table 1.** The correspondence between the numbering of the titin A-band domains used in this work (bold blue numbers correspond to A/I1-14 and A1-170) and the number of the Fn (red) or Ig (black) domain determined from the inferred complete gene sequence (NCBI:NP_001254479.2). See also <http://fraternalilab.kcl.ac.uk/TITINdb>

**Supplementary Table 2.** Chart showing similarities of A-band titin domains according to the work of Kenny et al [1]. D1-6 and C1-11 show the D-zone and C-zone super-repeats as seen in Fig. 1. The different colours represent groups of domains that cluster in the unrooted evolutionary tree. Cells with white background are Ig domains, most of which cluster together. Those underlined randomly cluster with non-A-band domains. The Fn domains are differently highlighted for different branches of the tree. The grey highlighted Fn domains have no specific association with a group. Note that the central 9 C-zone super-repeats, 2-10, have identical patterns. CSR1 domain10 (A52) and CSR11 domain11 (A163) differ from domains in the same position in other C-zone super-repeats.


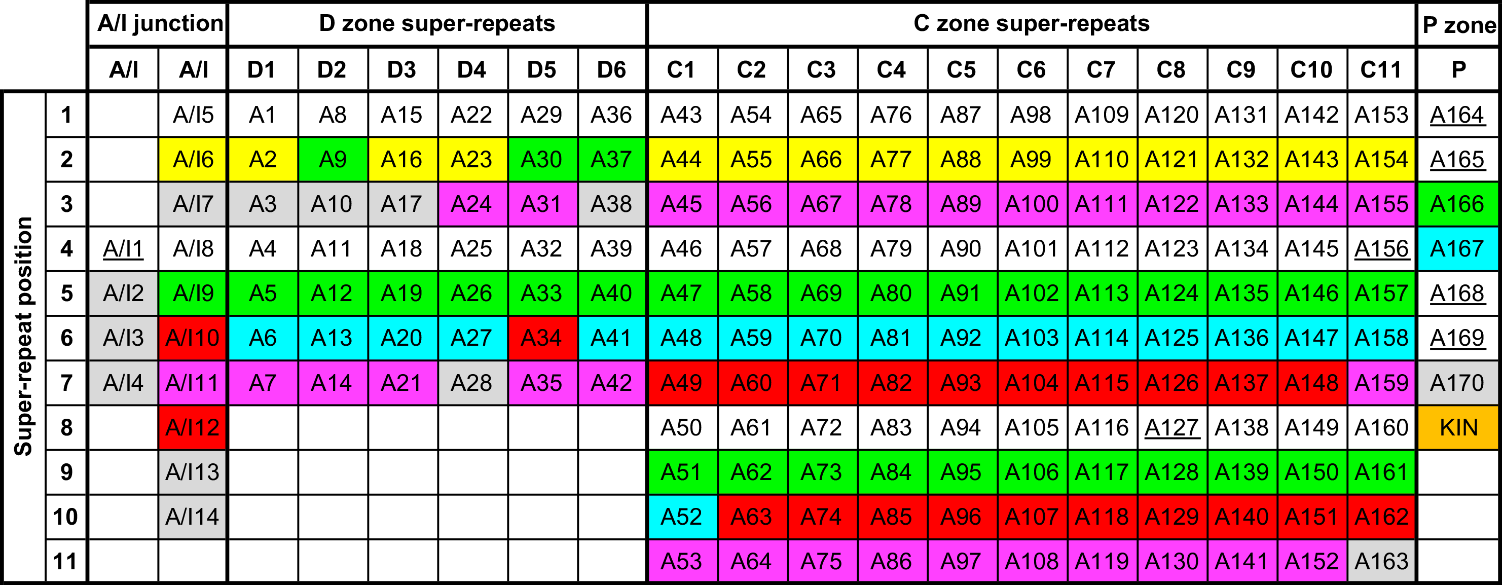


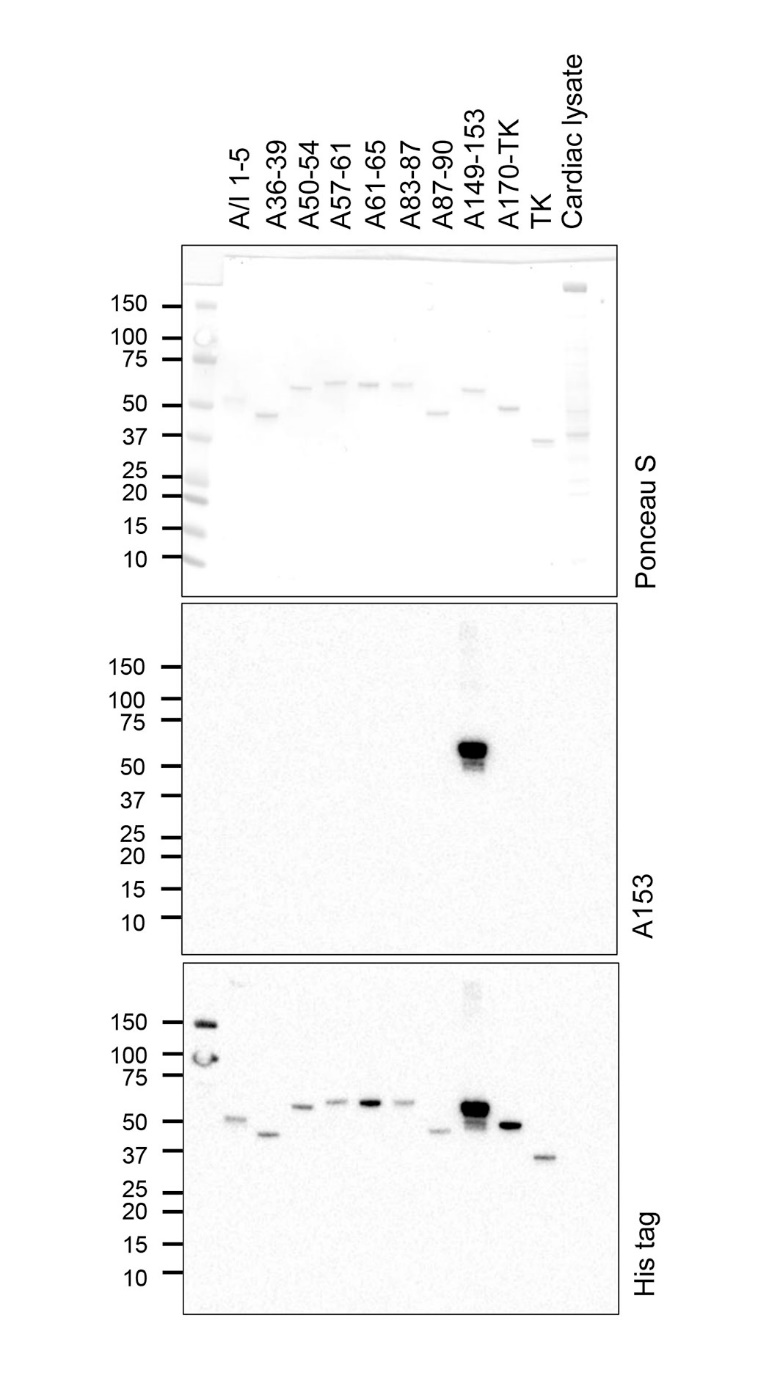


**Supplementary Fig. 1**. Western blots showing recognition of epitopes by antibody A153. His-tagged purified titin fragments and cardiac lysate (top) probed with the antibody A153 (middle) followed by probing with an antibody against His tag (bottom). The positions of standard marker proteins in kDa is shown at the left side of the blots.


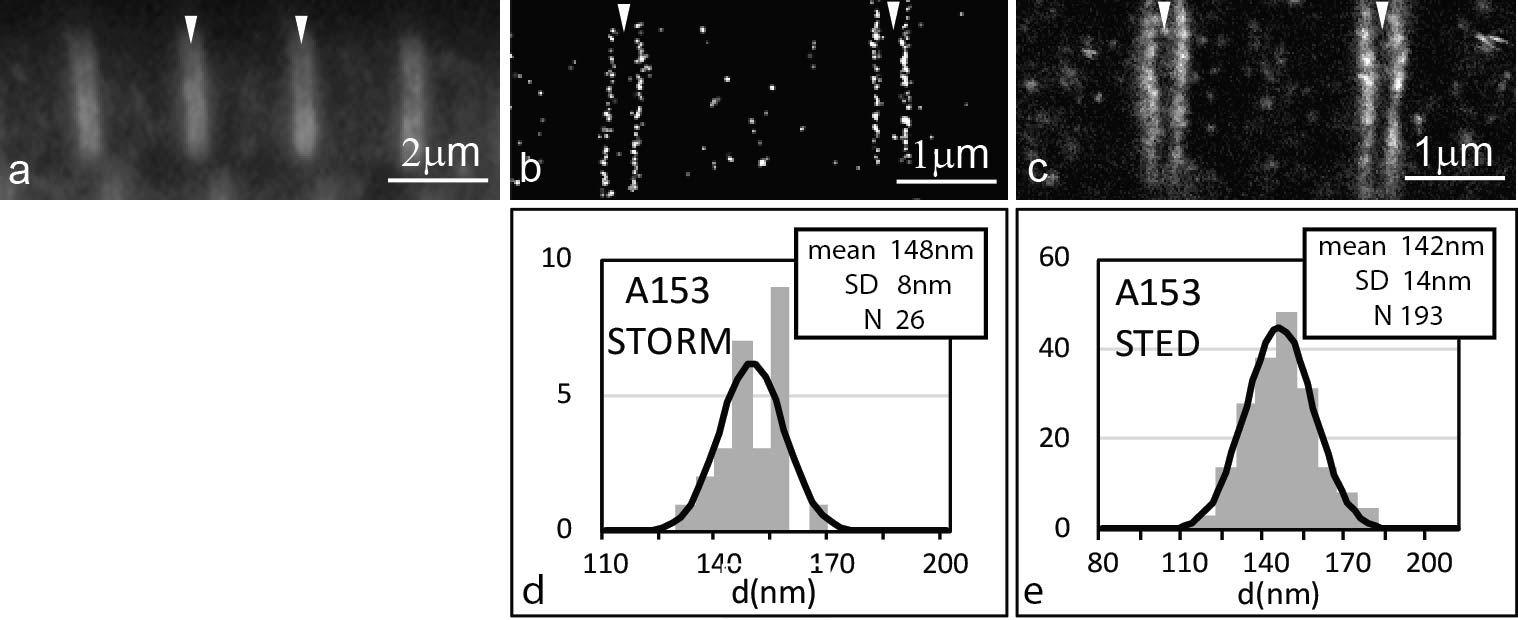


**Supplementary Fig. 2**. Rabbit psoas myofibrils labelled with titin A-band antibody A153 visualised with different imaging techniques. (a) Standard IF. (b) STORM. (c) STED. (d) Histogram of measurements from STORM images of distance of label from the centre of the filament. (e) Measurements from STED images. Arrows in (a)-(b) indicate the position of the M-band.


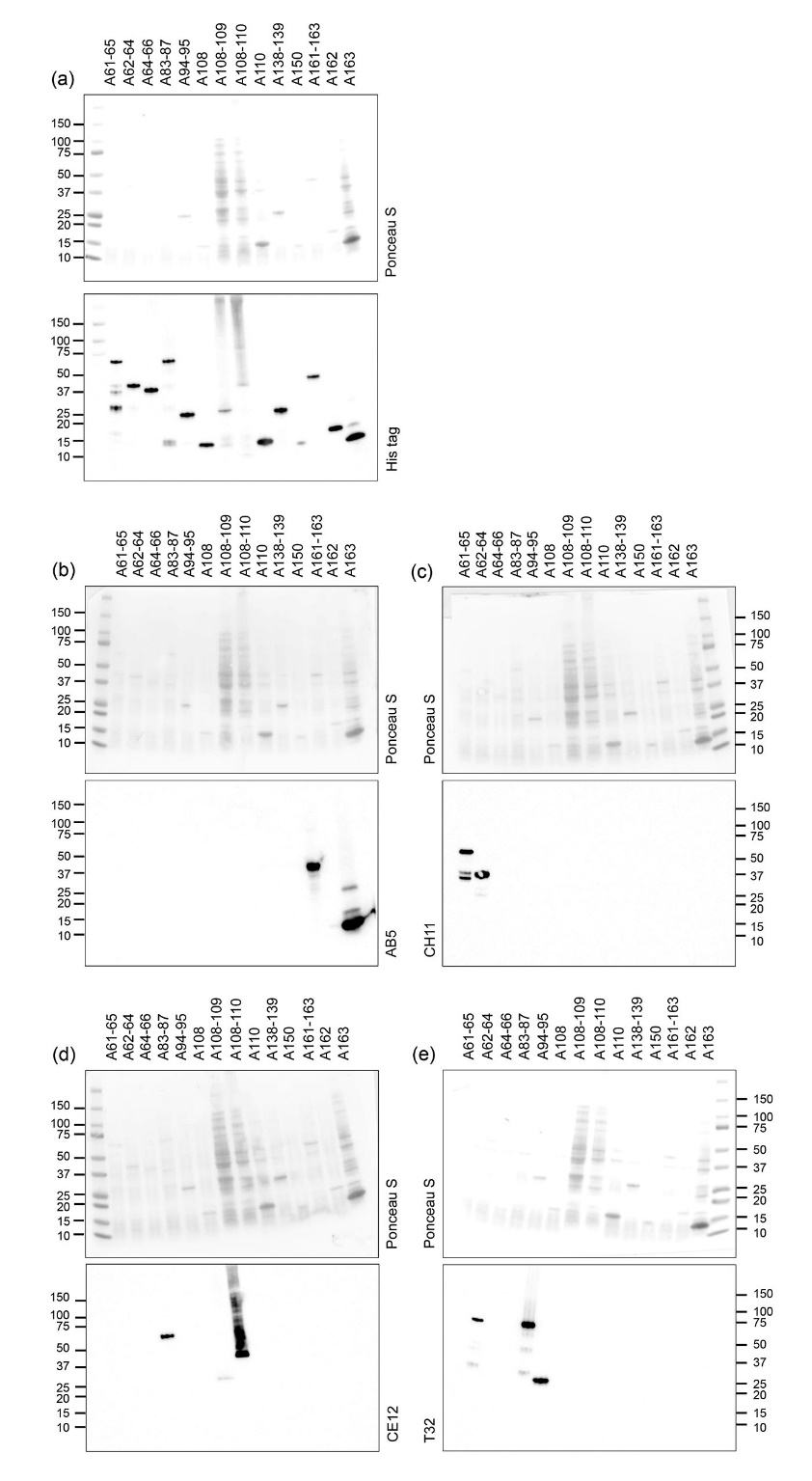


Supplementary Fig. 3 Western blots showing recognition of epitopes by titin antibodies. His-tagged titin fragments were expressed and probed with the antibodies a) anti-His, b) AB5, c) CH11, d) CE12 and e) T32. The positions of standard marker proteins in kDa is shown at the left or right side of the blots. Note that CE12 recognises two titin fragments, A83-87 and 108-109, separated by 2 C-zone super-repeats and T32 recognises three epitopes A61-65, A83-87 and A94-95 separated by 1 and 2 C-zone super-repeats respectively.


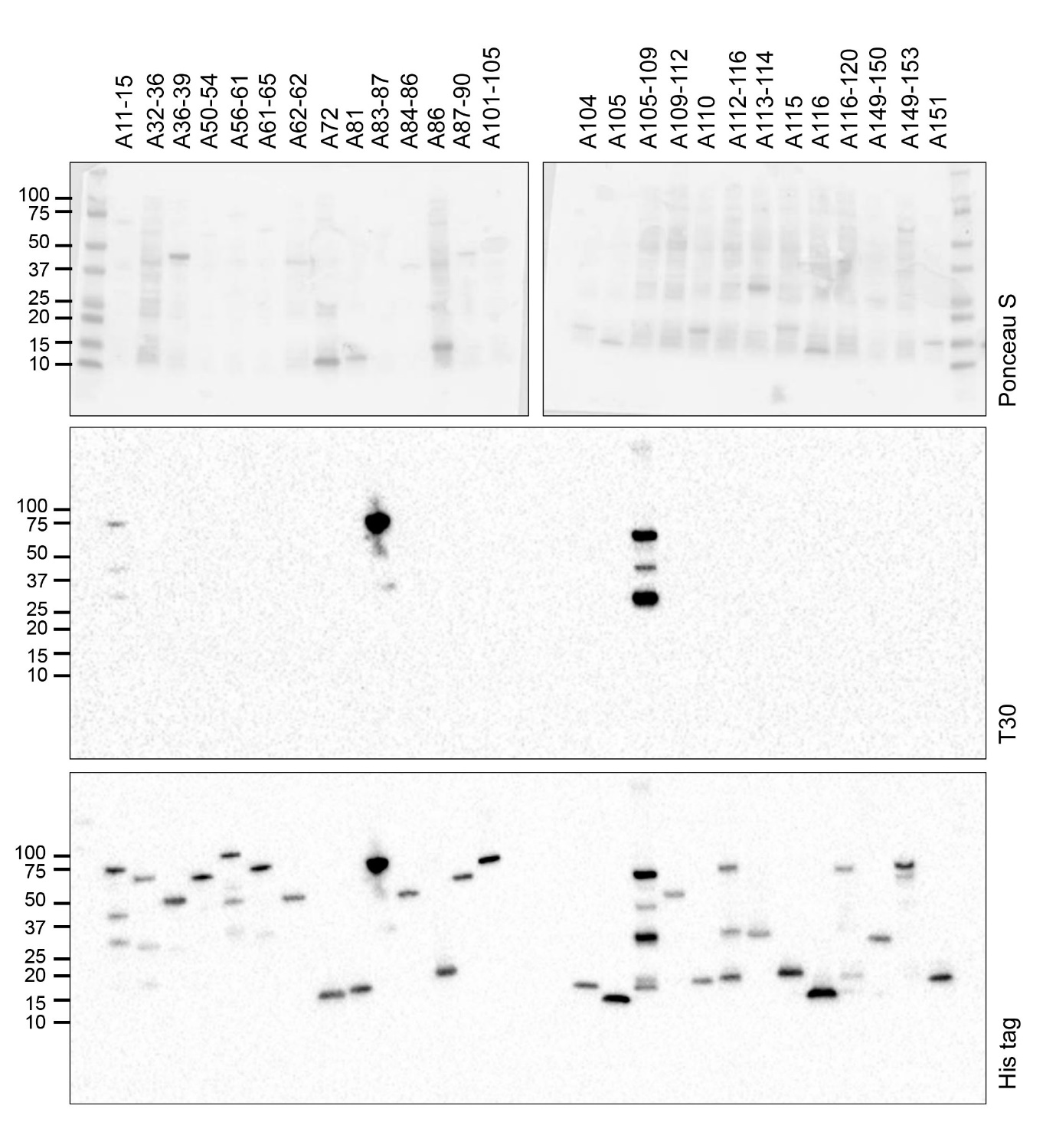


**Supplementary Fig. 4**. Western blots of expressed His-tagged titin fragments (top) probed with the T30 (middle) and His-tag (bottom) antibody. A84-86 and A105-109 are strongly labelled as predicted. Absence of label to overlapping sequences suggests that the border between A83-84 and A105-106 house the epitope for the antibody. There is also a weak interaction with A11-A15 which is not seen by immunofluorescence. The positions of standard marker proteins in kDa is shown.


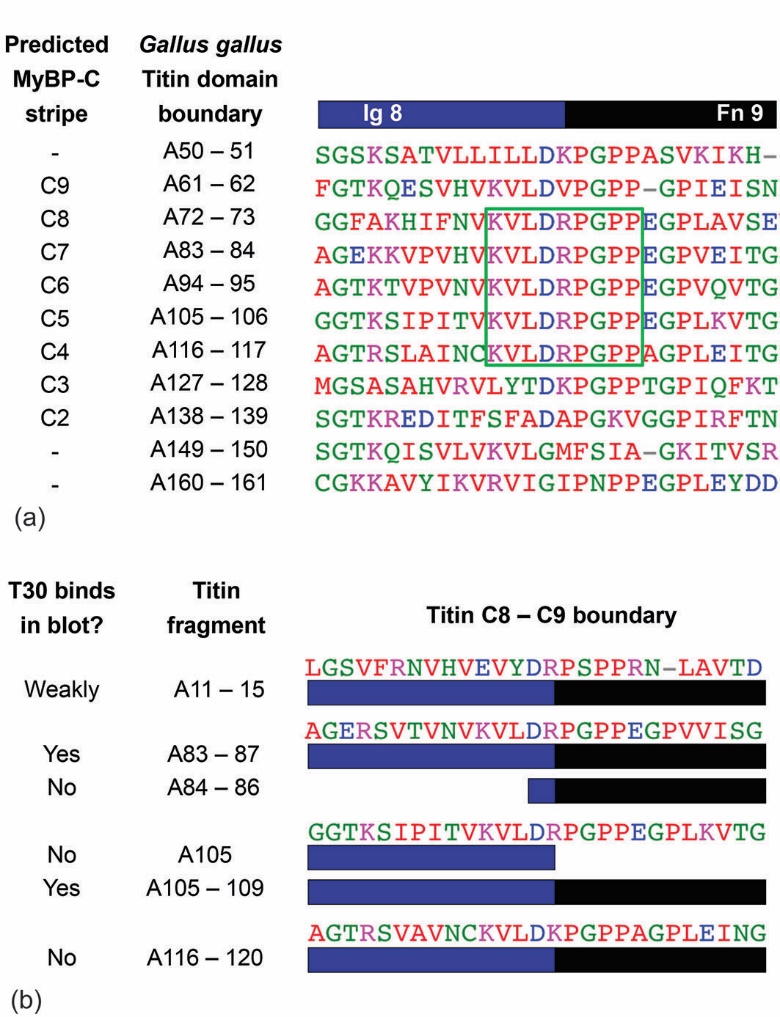


**Supplementary Fig. 5**. (a) Sequence analysis by Clustal of the linker region between domains at C-zone super-repeat positions 8 & 9 in chicken titin. Sequences were obtained from the chicken genome (GenBank: **BN001113.1**) Five of those sequences are identical (boxed) across the junction. These correspond by our reckoning to the positions of MyBP-C stripes #4-#8, the positions labelled by T30 a/b in the chicken [2]. This sequence is conserved in rabbit, mouse and human in four of the five cases. The exception being A116-117. (b) Summary of expressed human titin fragments in T30 binding regions which are or are not recognized by the antibody. Probing these fragments reveals that only when the boundary domain between Ig8 and Fn9 is intact does the antibody recognise the epitope.

[1] Kenny PA, Liston EM, Higgins DG. Molecular evolution of immunoglobulin and fibronectin domains in titin and related muscle proteins. Gene. 1999;232:11-23.

[2] Furst DO, Nave R, Osborn M, Weber K. Repetitive titin epitopes with a 42 nm spacing coincide in relative position with known A band striations also identified by major myosin-associated proteins. An immunoelectron-microscopical study on myofibrils. Journal of cell science. 1989;94 ( Pt 1):119-25.
